# The decision: Relations to oneself, authority and vulnerability in the field of selective abortion

**DOI:** 10.1057/biosoc.2014.39

**Published:** 2014-11-03

**Authors:** Sølvi Marie Risøy, Thorvald Sirnes

**Affiliations:** a Faculty of Social Sciences, Sogn og Fjordane University College, Røyrgata 6, 6856 Sogndal, Norway. E-mail: solvi.risoy@hisf.no; b Centre for the Studies of Sciences and the Humanities (SVT), University of Bergen, Allegt.34, PB 7805, 5020 Bergen, Norway

**Keywords:** selective abortion, prenatal diagnosis, abortion decision, state of emergency, authority, vulnerability

## Abstract

This article is about selective abortion. It concentrates on the existential, moral and social conditions that arise when pregnant women, using prenatal diagnosis (PND), are told that there is something seriously wrong with the foetuses that they are carrying. This is characterised as a micro state of emergency, where both normal cognitive categories and normative orders are dissolved. The analyses are anchored in the womens' own presentations and understandings of the processes and dilemmas related to the abortion decisions, and our most important empirical materials are interviews with women who have experienced them. Our main ambition is to show the relation between some important dimensions of the situation in which the abortion decision has to be made, and the special kind of authority on behalf of the women that presents itself. Of equal importance is the vulnerability of the pregnant women, resulting in a co-production of the women as both Sovereigns and Homo Sacer in the decision situation. We also analyse some of the experienced relations between the women and the foetuses, and how the women constitute themselves as moral subjects, with a particular emphasis on the motifs of sacrifice and self-sacrifice. It is a central argument in the article that we have to understand the specificity of the decision situation, without reducing it either to other phases (before or after) of the total processes of PND and selective abortion, or to general discourses of disability or normality. The specificity of the situation in which the abortion decision is made is a pivotal point in society's regulation (in a broad sense) of the field and in the constitution of the regime of selective abortion.

## Introduction

Prenatal diagnosis (PND) and selective abortion are closely connected. The majority of those who exhibit serious foetal defects decide to abort, and many have noted that PND contains an institutional expectation of selective abortion. Nonetheless, the actual decision remains an unexplored area in presentation and analysis, with a few exceptions. The direct conclusion from diagnosis to result (that is, whether or not to abort) misses the central process where the most essential effects are produced. The state in which the decision is made is a pivotal point in our prenatal diagnostic regime. We cannot understand society's relation to selective abortion without studying the logic of this decision.

We deliberately use the word ‘decision' and not ‘choice'. The latter carries connotations of a moral philosophical debate based on idealised situations, which are far removed from the real world. Instead, we want to analyse the phenomenology of PND and selective abortion. We will not evaluate the thoughts and reasons of the pregnant women concerning selective abortion on the basis of theoretical, ethical perspectives. This does not imply that we have no normative intentions. They are different from and more relevant than imagining and judging ethical validity claims, an activity which in many ways has come to a dead end. If we make some steps to understand the decision as a practical empirical event, we may be able to develop a critique of how its diverse conditions produce decisive effects. Even a small contribution in this direction will be of vital normative importance.

We will start with the situation when a pregnant woman finds out that there is something seriously wrong with the foetus. The analyses are anchored in the women's own presentations of the processes and dilemmas. On the basis of the stories of individual women, we will analyse the existential, moral, social and political conditions of the decision-making process around selective abortion. Even if there is both dissonance and continuity in the presented stories, we maintain that it is possible to develop a central social and political logic from the individual narratives.

The article is not about the decision process related to so-called ‘blind abortions', where the women do not have any specific knowledge about the conditions of the foetuses. We concentrate on selective abortions, and the women in our material really want a child, the pregnancies are most often planned and their social and economic situations do not make the arrival of a new child problematic.

There is another qualification which is even more important. We will not be exploring the discourses of disability in society. In many of the individual stories in our material, the foetus will have no chance at all to survive a birth. In these cases, the link between the decision process and the general conditions of disabled people will be very hard to establish. When the newborn child may not only survive but live for many years, the connection will be important to analyse. Intuitively the political, social and cultural discourses and conditions of disabled people may constitute a vital background for the decision processes in such cases.^1^ However, we resist a direct and automatic import of the general logic. First of all, the specifics of the situation (after a serious defect in the foetus is discovered) must be analysed and understood. Only then can we start the hard and cumbersome work of establishing the links between the selective abortion decisions and the general discourses and conditions of disability. There might be some surprises. That is the nature of empirical work. There is a possibility of very paradoxical relations, as well as of the discovery of a decision logic that cannot so easily be reduced to the dominating disability discourses. One of the main objectives of the article is to contribute to establish a necessary foundation for the discussion of these relations.

We also want to clarify the empirical scope of the article. The topic is neither the decision processes leading to the engagement with prenatal diagnostic technologies, nor how the women present and explain their abortion decisions to their families, friends and other relations *after* the abortions. These are different phases, possibly involving very different logic. Also on this point, we resist a reductionist approach. Neither the before, nor the after, may directly explain what is going on in the critical situation when a selective abortion decision is made.

Since the other two phases are also parts of our total research material (Risøy, 2010),^2^ we will only briefly indicate that they are governed by social logic that are more compatible with the general disability discourses, as well as the ‘normal' hierarchical relations between medical experts and patients. The health system and medical apparatus perform with their normal authority in the early phase and recommend various examinations to pregnant women, but there is a strong undertone of something else when genetic counselling is offered. In such cases it is communicated that anything is a possibility in PND, and if the women find themselves in a situation in which defects are detected, the doctors will no longer be able to tell them what they should do. This is a break with regular medical practice. Diagnoses are normally followed by authoritative advice on treatment, if available, but during counselling there is a warning of the retreat of medical authority. Future situations are indicated in which there will be no normative guidance,^3^ and the pregnant women must therefore be prepared for something out of the ordinary.

## The Field of Research

Although medical research on PND and selective abortion has been concerned with the choices that a woman faces, genetic counselling, various conceptions of risk and women's reactions after selective abortion, it is often quantitative and does not probe very deeply into personal experience. The social and political aspects are only superficially present (Salvesen *et al*, 1997; Grimes and Snively, 1999; Getz and Kirkengen, 2003; Kersting *et al*, 2004, 2005, 2009; Burgoine *et al*, 2005; Keefe-Cooperman, 2005; Korenromp *et al*, 2005a, 2005b; Getz, 2006; Skari, 2006; Offerdal, 2008). The work of philosophical ethics often says little about those practical morals which guide the actual concrete decisions that are made on selective abortion, or what connections these decisions can have to social and political regimes (Tännsjö, 1991; Singer, 1994; Purdy, 1996, 2001; Habermas, 2003; Solberg, 2008).

The social science and humanistic studies of the public and political debate on PND and selective abortion (Bakshi, 2000; Flatseth, 2009) intersect with our study. However, it cannot be taken for granted that the same discourses dominate politics and praxis.

Rothman (1986) and Rapp (1987, 1999) were pioneers in social science studies of praxis. Rothman's concept of “tentative pregnancy” led to later perspectives on PND, genetic counselling and risk assessment (Heyman *et al*, 2006; Samerski, 2006, 2009; Schwennesen *et al*, 2008; Schwennesen and Koch, 2009; Kato, 2009; Gupta, 2010; Ivry, 2010; Harris *et al*, 2013; Markens, 2013). Rapp showed how women in this field came to be constituted as moral pioneers. It became a woman's responsibility both to explore the variances of different perspectives of normality and also to make decisions. Although some of the dilemmas regarding PND have resonance in the decision process of selective abortion, the two situations are ontologically different. ‘What if' will always be different from ‘happened to me'.

There has been some recent social science research focusing on the decisions of selective abortion. McCoyd (2007, 2008, 2009a, 2009b), as we ourselves have done, looked at the whole process that the woman goes through, from the choice of diagnostics to the grief afterwards. McCoyd (2007) describes how “good mothers” do not have “bad” babies, and that there exists a cultural contract in pregnancy which is broken if one consciously brings a child with defects into the world. Selective abortion in Vietnam (Kato, 2009; Gammeltoft, 2014) and India (Gupta, 2010) has been the focus of recent research.^4^ Gammeltoft (2014) shows how reproductive choices in Vietnam are shaped by a concept of belonging rather than the individualistic concept of freedom. These studies show how selective abortion decisions are shaped by general social and cultural logic. In contrast, we analyse the exceptional nature of the situations in which these decisions are made, and thereby we show the limitations of normal social order and discourses.

## Legal and Institutional Context

In Norway, there is a routine offer of an ultrasound examination in weeks 17–19, which is accepted by almost all pregnant women. Although it is not obligatory, the examination is perceived as such by most women. There is a legal distinction between ultrasound as prenatal care and diagnosis. The difference lies in the intention: is it to search for defects (PND) or to do a ‘follow-up' on the pregnancy (prenatal care)? The examination in weeks 17–19 is legally defined as prenatal care, and as such no genetic counselling is given before it. In spite of the legal status the quasi-obligatory examination is used to search for and identify defects, and the majority of them are in fact detected in this way. The result is a combined legal, social and psychological paradox: the routine ultrasound, which was neither defined nor perceived beforehand as a prenatal diagnostic, is perhaps its most important tool. It is primarily women aged 38 years or more at the time of birth, or who have special indications, who receive a direct offer of double and triple testing^5^ and invasive methods such as amniocentesis and placental biopsy, as well as ultrasound with the specific intention of detecting defects. Of course, if there are indications of possible defects in the examinations during weeks 17–19 then the women are followed up by the medical system and are offered the same kind of tests. Genetic counselling is given before these targeted examinations.

In most cases, women must decide whether or not to terminate the pregnancy once defects are detected. It is only in rare cases that the foetus can be operated on while still in the womb or directly following birth.^6^ In Norway, abortion is based on free choice up to and including the 12th week of pregnancy. After this, access to abortion is regulated by committees and the Abortion Act §2 c, which is called “the eugenics paragraph”, becomes relevant. It states that abortion can be performed when “there is a great danger that the child can have serious illness”. In practice, the committees almost automatically give permission for abortion when a woman applies on the basis of foetal defects. In reality, there is also a free choice of selective abortion after the 12th week. The Act places an absolute limit on termination at viability. As a consequence, there is no time limit for abortion or induced labour if the foetus is not viable because of the defect.

## Our Study

In the study, we undertook a comprehensive examination of different aspects of women's encounters with PND and selective abortion. We carried out observations of laboratory work, genetic counselling, ultrasound examinations, and the collection of amniotic fluid samples and placenta biopsies. The most important data are the interviews. Fifteen interviews were conducted with doctors, priests, midwives, administrative employees, social workers and genetic counsellors at the hospital. Twenty-three interviews were conducted with women who have had experiences with PND. Eight of them had amniocentesis performed without any defects being detected, whereas 15 women did show defects in the foetus. Of the latter, 12 decided to have selective abortion, whereas 3 decided to carry to term despite serious defects. These informants were chosen through a process in which the main objective was to achieve variation and breadth of diagnoses. We wanted to include both borderline cases, in which there can be disagreement over the severity of the PND,^7^ as well as different types of terminal defects. The women were not recruited either through hospitals or the health system, but rather by placing advertisements on Websites and Web forums. This has both pros and cons methodologically. The recruitment is based on a self-selection process in which those who were motivated to speak about their experiences made contact with the project. At the same time, the women did not associate the interviewers with the professionals with whom they were in contact during the process of PND and selective abortion, and the project avoided being tainted by the authority structures which dominate the health system.

In our study, we have explored all the phases that women go through, from the first ultrasound examination through to the grief after abortion. But the article focuses on the situation *after* defects are shown in the foetus and *until* a choice is made concerning abortion. The theme is the abortion decision. We want to analyse how the women experience the decision situation, and therefore we concentrate on the interviews with the women.

The women were interviewed some time after the actual processes. There are several reasons for this. First, we recruited women who wanted to talk about their experiences in retrospect. Second, the ethical principles left us with no other choice, as we did not want to influence and affect the women in their most critical and vulnerable situations. This was a deliberate strategy. In addition, the nature of the ethical regime in Norway makes such interference nearly impossible. This research strategy has, however, some methodological implications. For example, there may be some distortions of memory and retrospective rationalisations. On the other hand, the different phases of the women's experiences, and especially that which is the topic of this article, obviously made a very strong psychological impact. During the interviews the women re-experienced some socio-psychological conditions which were very hard to forget, and to a large degree they seemed to relive the phenomenology of the decision situation.

We will now address the situation that arises when something wrong with the foetus is detected.

## The Shock

*Reidun* and *Mette* dramatically recount the “obligatory” examination in the 18th week of pregnancy.

So we came in and lay down on the table and she explained what they were going to do (…) Then she made a jerk with her head, and she got very quiet. I thought that this isn't good (…) So I thought, no, no, don't get hysterical. Then she said: I see here that there is something wrong. And so the whole world just collapsed, really. Reidun, abort, Hydrocephalus

Something dramatic is foreshadowed when the midwife becomes quiet. The silence signals the dissolution of the logic of the examination. This is no longer ‘prenatal care' but ‘PND'. Subconsciously this possibility must have been present, as the dark emotions start flowing even before the midwife says anything about the foetus. The quote then presents the theoretical challenge of our research: we must try to understand the social dimensions and implications of the total collapse when ‘something wrong' is detected.

So it was just routine, you know? We were at [name] hospital and they are probably not so good [with] ultrasound: “See, an arm”, “see, a leg”, and so on for about 45 minutes, then it was suddenly: “Oh”, there was this sack filled with fluid on the baby's neck. And then it was full speed into [name of hospital]. So it came as a shock. Mette, abortion, Mosaic Down syndrome

There was a strong contrast in the situation between before and after the discovery. The following shock makes the calm state characterising the examination (routine) look naïve, and the new severity of the situation is expressed both by the change of pace and the mobilisation of better medical competence at the new hospital.

“In the beginning of December last year, we had an ultrasound check-up and were very happy. We were a little naïve, but had no idea that something would be wrong”, begins *Kjersti's* narrative. This is what happened when they returned for the next ultrasound examination:

I asked the midwife if the water on the brain was gone, since she said the last time that the baby had water on the brain, but that it was normal when it wasn't so big. She said “not completely”. Then I began to get a bit uneasy, but not so much. The same doctor as the last time came in and they began to tell me what was wrong. The baby had an umbilical hernia, but it could be operated on, so I didn't take it so hard. Then they found something wrong with the kidneys and worst of all, the head … After this, everything was just chaos in my head. I sat there with [my husband] and the midwife and she said in a nice way that the baby had a number of problems, but that she would send us to [a larger hospital] because they had specialists there. But I didn't understand why I should go there. I was over the time limit for abortion (21 weeks) so I said that. But she said: no, this is a special case, so … don't remember any more. She held me with tears in her eyes, a wonderfully fine lady who I have talked to since also. They made an appointment at [the larger hospital] for the next day and we went home. Can't explain what we were thinking. It was just unreal. Kjersti, abortion, Trisomy 18

The beginning of the process was marked by the same retrospect naïvety, and even the water on the brain that had been detected during the first ultrasound did not cause any great insecurity. But her unease increased steadily until the problems with the kidneys and brain created emotional chaos. The medical staff's most important contribution was the attempt at sharing this condition and trying to break the loneliness of the situation. Kjersti did not realise that the defects had removed the foundation for the normal legal regulation of abortion, and that the 21-week limit was therefore no longer valid. Essentially her experience cannot be communicated. The situation is overburdened with the inexplicable. This characterises the new, dramatic subject position of a woman who is pregnant with a baby with which something seriously wrong is detected.

Even if prenatal diagnostic examinations such as amniocentesis or placental biopsy are understood as a search for disabilities, finding what they are actually searching for is beyond imagination. *Mona* expresses this paradox:

I live healthily and have borne four healthy children, and have become pregnant immediately. So I didn't imagine that it would … It was just for safety, for safety's sake (…) I was most excited to find out the sex, that was what I was waiting for (…) Well, no, I got an absolute shock, of course. It was beyond the pale of what I had expected was going to happen. Because there are so few, it's so unlikely … . Mona, abort, Down syndrome

The fact that Mona had experience with amniocentesis from earlier pregnancies without any defects being detected, and also the fact that she had on four occasions proven her reproductive capability, was important for her expectations of the test. The difference between before and after the discovery – “just for safety” and “absolute shock” – could not be greater. In addition, earlier priorities vanish. Although she had a desire for knowing the gender, the diagnosis totally overshadowed this question; the fact that the foetus was a girl was not mentioned until a long way into the interview.^8^ Statistics, which had probably given her a false sense of security, afterwards added to the extraordinary nature of her situation.

*Charlotte* knew that she was a carrier of Fragile X syndrome and did not wish to bring a child into the world that was either a carrier or affected. She was therefore active and had a placental biopsy performed. After some waiting, they received the message that everything was fine, and that they could enjoy the pregnancy. But then this happened:

She called me. (…) And so I smiled a little and said that “Now, you shouldn't scare me. I'm getting some bad vibes here”. And so she says that “Yes, unfortunately, I have to give you some bad news”, And as you can imagine, I fell apart a bit. It was affected after all. (…) My husband was in the neighborhood so he got there in about three minutes … and so we talked about it with each other and decided that we would have an abortion as soon as possible. So we talked to [the hospital], and they say that, this was on a Thursday, they say that “You can come on Sunday and have it done that evening”. So we tell them that this is not satisfactory, my husband says: “because we want it done now, and you just need to make it happen”. Charlotte, abortion, Fragile X

Charlotte was in her 16th week when she received this information. She goes back in time to the moment when she took the telephone call and describes her feelings: “It was, of course, chaos. Then it was: is this possible? How can you get a message like that? How is it possible to be so wrong? Can they treat people in this way? Anger at the hospital”. The shocking experience that Charlotte underwent was actually the hospital's mistake. She made the conscious choice to search for defects, with the clear intention of terminating the pregnancy if they were found. But the medical system failed her. Even though she was prepared for finding Fragile X syndrome in the first phase, the false test result had calmed her and mentally established a normal pregnancy. In the next phase, this is transformed into chaos and a breakdown of meaning.

## Facing Normality and Deviation

Ultrasound provides a meeting with the foetus. It becomes humanised and incorporated into the family.^9^ The ultrasound picture and the explanation provided by the medical personnel ‘produce' the human. But what is produced when the examination detects defects in the foetus?

We certainly saw the little heart beating, and the beat was clearly completely healthy. And sucked on the thumb and, or in any case, scratched itself, now I have heard that they don't suck their thumb at 19 weeks. But it was a fully perfect little baby. (…) But there was no defect on the outside. But much was missing; she had almost no lungs. Because with Triploid syndrome it's like the body doesn't think it has the resources to develop everything, so it only does the most important parts – the brain and heart. So it drops, for example, the kidneys. They are certainly of no use anyway, not yet, nor the lungs, since they are not so necessary in the belly either. So, the way it was: the lungs were teeny tiny, the kidneys were almost not there. And a lot of intestinal blockage. And webbed fingers, the ring finger and middle finger on the second and third joints. (…) And the feet were like small fans (…) They took a long, long time explaining it. About this and why it was this way and showed again and again, and we asked to see more of the profile, I wanted to see more. And they really took a long time. I mean, a long, long, long, long time. I thought it was an eternity. Sara, miscarriage, approved abortion, Triploid syndrome

Apparently, Sara first points out the normal (the baby sucks at its thumb) and then the defective. But with a closer reading, it can be seen that the whole quotation, in fact, concerns a “perfect little baby”, which has shown a very specific kind of survival instinct. Just as a fish has adapted to its life in water but not on dry land, the foetus has adapted itself to a life in the womb but not outside it. The fact that the heart was healthy emphasised the foetus' staying power under its current conditions. At the same time, it lacked several organs vital for a separate, individual life. It is as if during pregnancy the foetus prioritised the relationship with the mother to the extent that it could not survive outside her body. As a consequence, the relationship became extraordinarily privileged. The ultrasound, showing all the terminal defects, did not create any distance, but in fact a very special sense of intimacy between the mother and the foetus. Sara constantly “wanted to see more”, as if the missing organs intensified the material and emotional bonds between them. The time dimension and sheer patience of the medical personnel therefore became very important. The long duration and even all the repetitions of the examination process were very precious – much more than at ‘normal' ultrasounds.

I wanted to lie here the rest of my life. (…) A necessary eternity. To be able to just lie there and lie there and look at the heart that just beat and beat (…) it was very good that so much time was taken. Sara, miscarriage, approved abortion, Triploid syndrome

In this context it is worth remarking that receiving the initial message that something was wrong with the foetus represented an extreme burden for Sara: “It was as if everything disappeared. It was completely empty. So horribly painful at some point inside the pain”. On the basis of this pain, she established the image of a baby which was ‘perfect' in its own right.

The next case, *Reidun*, provides a striking contrast to what we have just seen:

I understood that this was very serious, and we knew there wasn't much hope. I listened half-heartedly, but when they went through and saw that something was good, they certainly said that too. And I think that was a burden, I thought the more that was positive, the worse it would just be. *You had begun thinking about the choice?* Yes, since he had said so much that I understood that … (…) it had a large amount of water on the brain and outside, between the skull and skin and down the back and in the stomach, it was enough for me to see that this is incompatible with life. And so, they began to say that he has five fingers and five toes and: “Forget about that”, it's not important. So … (…) I said that “I can't take any more now, I want to be done here now.” And so they finished up pretty quickly then. Reidun, abortion, Hydrocephalus

In both cases, the defects of the foetus are incompatible with life and led to decisions to have selective abortions (although a miscarriage preceded it in Sara's case), but their effects on the mothers were completely different. Although they were interpreted as an expression of a very special bond between the mother and the foetus in Sara's case, they led to dissociation in Reidun's case. The non-relation is, however, threatened by the normal features, which confuse the entire situation. Pointing to five fingers and toes is a humanisation of the foetus. Whereas Sara embraced the humanisation of a foetus which could not live after birth, Reidun not only deliberately rejected it, but perceived it as a serious burden imposed on her by further communication. This also led to a contrasting importance being placed upon time. While listening to the medical personnel was a consolation and a means of coming to terms with her destiny for Sara, it increased the vulnerability for Reidun.

Our third case in this section, *Guri*, represents yet another kind of reality. After she had been fully informed by the doctor of the defects affecting the child and which possibilities were open to her, she went home.

The very first thought I had, when I went to bed at night, I thought, “Oh, God, I have a monster in my belly”. I have seen films, you know, invasions from outer space and terrible things. Some slimy thing with long arms, it was horribly brown and ugly and just suddenly exploded out of my stomach. Or just ate me up from inside. That was how I thought about this human in my belly, the first night I went to sleep. I just thought, get rid of this belly. I didn't want it there, take it away, take away this monster. Guri, abortion, Terminal heart failure

We have neither the privileged relation between the mother and the foetus, as in Sara's case, nor the vulnerable non-relation as in Reidun's. Here, the foetus has become a horrible threat to the mother or a deadly enemy. Although she does not make a direct reference, it is obviously the world of the ‘Alien' films from which Guri takes her associations. In the first film, the crewmember Kane is invaded and the alien grows inside him until the brutal ‘birth' takes place, during which the alien more or less ‘explodes' out of his stomach, immediately becoming a mortal threat to the rest of the crew. Guri became pregnant after several IVF attempts. It is possible that this genesis of the pregnancy amplified the mental connection to the Alien films.^10^

While Reidun did not want to think or know anything more than absolutely necessary about the foetus, both Sara and Guri indulged in details, which seemed to be very important for them. There is a certain beauty in Sara' description. Guri, however, expresses an extreme aesthetics of the ugly and monstrous. Sara admires her foetus, whereas Guri is full of repulsion against what is inside her. There is also a very opposite sense of belonging. Sara's child belongs so much to her that it is not able to live outside her body. Guri's foetus does not belong to her at all. It is an alien. Therefore the pregnancy is not the growth of a connection, but an invasion. Whereas Sara's case expresses an almost too perfect harmony, which makes birth and biological departure impossible, there is a deadly fight going on in Guri's. Therefore she had no choice: the foetus-enemy had to be aborted. Guri tells us that the child for which she had been so glad, and for which she had already struggled so much, had now taken on the extreme opposite meaning.

After defects have been detected in the foetus, there is a fundamental decision to be made. Should it be aborted or not? We have given a picture of a condition characterised by shock and pain.^11^ An important dimension is also the feeling of a break with reality: “This is just a mistake. Soon we will be in the real world again” (Sara). “I began to cry and it was just as if I was looking down on us from above. Exactly like a film or a nightmare” (Kjersti). It is in this type of situation, in which there might even be a thin line separating reality from dark fantasies, that the important decision must be made. The combination of these elements can be summarised and condensed by a quote from Sara: “It was a state of emergency”. Even if we are not going to infuse any theoretical intentions into her use of words, there is an important clue here. In political philosophy, ‘state of emergency' indicates a situation in which the basic, normal conditions that are presupposed by social and juridical order do not exist. There are situations that are too extraordinary for rules to apply. Although these kinds of perspectives are mostly employed on the state level, we think that they might be productively transposed to the micro-situation of the abortion decision. We are fully aware that this jump from one level to another might cause some problems of interpretation, but there are some obvious reasons for it. The decision situation is potentially dense with juridical aspects, as well as normative prescriptions constructed on a social and political level. It represents a condensation of general moral perspectives. In the micro-state of emergency, however, they lack the silently presupposed basis for being relevant. “ … like the outside world just stopped. Everything else is so unimportant. It is only inside that room that things happen and everything outside doesn't exist” (Reidun, abortion, Hydrocephalus i.a.). In this context, we may tentatively interpret the norms and rules as belonging to the ‘outside'.

## The Decision: Authority

Following this chain of thought, the next essential question is: Which kind of authority is present, or rather presents itself, under these conditions?

(…) but it was as [the husband] said, it is you who have known life, it is you who carries it, it is you who will protect it with your body, no matter what I say or do, support you in or not, it is you who has to shoulder this. And that is not something anyone can change. And it is true enough, he can [not] go in and make a choice for me, since it is up to me. And I was also concerned with protecting his feelings and right to be a part of this decision … so I chose to include, or give him the feeling that you also decide, because it was important to him. But it didn't make it any easier. Vilde, abortion, Klinefelter syndrome

The woman presents herself as the unchallenged decision maker. She expresses this on behalf of herself and her partner. The man realises and admits that he has no given position, but can be included by the woman if she so chooses. But a shared decision is not a reality, and is more of an attempt to give the man “the feeling that you also decide”. The attempt to give others responsibility therefore fails, as does the desire to be relieved of the burden of the decision to terminate: “But it didn't make it any easier”.

*Jorunn's* boyfriend did not want a child with Down syndrome. But his reservations concerning having a “mongoloid boy” makes Jorunn realise that it is her decision whether or not to carry to term:

No, he didn't think it was ok. He thought it was fine if we had a baby, but he didn't think it was so great to be a pappa to a … mongoloid boy (…) So I thought I had to decide for myself …. Jorunn, birth, Down syndrome after amnio

*Linda* had an abortion after terminal defects were discovered. It was her experience that those around her wished to influence her decision: “But I actually felt I was pressured a bit, both by my husband and, he was certain that he didn't want me to go out”. She also felt pressure from her mother:

No, [my mother] she couldn't take it at all. My sister said, before we had decided, that my mother had said that “I can't handle this if [Linda] goes around in late pregnancy with a child that cannot live”. She couldn't handle it. And it affected me and, while I didn't think that it should affect me, since it was me that should decide what I was going to do, my sister had said to her that [Linda] has to find it out for herself, you can't tell her … she meant that it had to be my choice and, yeah, not to mix yourself up in it (…) But I felt pressured, I did. I had actually made the decision, and so called a doctor, before we went back. And so then she said that “It is your choice” (…) In one way, I wanted her to say “Clearly, you have to do it”, you know. But she said “It is your choice”. And she wouldn't say what she thought about it either. *Did you ask her?*

Yeah, I did. Linda, abortion, missing kidneys

The influences are both direct and indirect. Ironically, it is Linda's sister who both conveys the mother's pressure and balances it with her own evaluation of the situation, confirming Linda's sovereignty. The message is paradoxical. There is strong advice about what to do, with the mother (or sister?) using her own mental health as a pressure mechanism, while the very relevance of the advice is undermined. The subjective effect on Linda is two-fold, both weakening and strengthening her position. With regard to the doctor she is seeking a way to escape responsibility. When Linda calls a doctor she “in one way” wanted her to say “Clearly, you have to do it”. But this was after Linda had actually decided to have an abortion, and therefore concerns a confirmation of the decision rather than a release from it. The quotation does not give the impression of a woman swinging back and forth, first following one view and then another, but in fact shows a woman in the midst of a duality, who stands alone in the decision to terminate. After all, she is the one who, in spite of the pressures, has the position and the authority to combine the factors.

I knew that I would never have an abortion, myself, I would not have managed it, but (…) I have a friend now who has just had an abortion. She (…) chose, after some tough rounds, to have an abortion. And she was afraid to tell me about it, but I said to her that “I respect … so much of what you have done”. We all get knocked around here in life, every one of us, “we have to make tough decisions” and all choices demand equal respect. So I would never judge a person who had an abortion, either because it wasn't a good time to be pregnant, or because they had learned that it was going to be a child with Down's syndrome (…) I think it is equally brave to make that decision. Even more so. *Yes … it … can affect the rest of your life.* Now, to have had our son would have affected the rest of our lives also, but I see it as a much more positive effect than having to live with such a tough choice that I know I couldn't live with. Gerd, birth, Down syndrome after amnio

Gerd gave birth to a child shown during pregnancy to have Down syndrome. Not even for her is it possible to criticise the decision to abort a foetus with a similar syndrome. Gerd cannot evaluate other women's decisions, only her own. It is only possible to respect the choices, whether they concern “blind” or selective abortions. The fact that the decision is “tough” and will “affect the rest of your life” creates an exclusivity that nullifies external evaluation criteria. The word “brave” indicates the women's short- and long-term vulnerability, which in the end can be shared by no one else. Only the pregnant woman may judge what it is possible to live with. This is true no matter how the life is affected. It is about pregnancy and birth as existential events and conditions primarily affecting only the woman. Everyone else has only a secondary relationship to them, and therefore external judgments are not an alternative. There is an indisputable authority that presents itself in the decision situation, which is closely linked to and enhanced by the woman's exclusive vulnerability. As a consequence, the abortion decision is individualised.

## The Decision: Reasons and Dilemmas

We have tentatively characterised the condition under which the abortion decision is made, as a micro-state of emergency where the vulnerable woman shows herself to be the sovereign. As we have indicated, this does not mean that the situation becomes a cognitive and normative ‘black box'. There is a gap of understanding between the pregnant woman and the rest of the people and actors involved, which partly accounts for her exclusive authority. At the same time, there is a dense communication unfolding on many levels, both concerning concrete bodily and psychological experiences and normative considerations. The women have many perspectives on the reasons for their decisions, as well as their dilemmas. We will expand on some of these. First of all, we will enter the situation in which the defects revealed by ultrasound are incompatible with life.

### Incompatible with life

She told us which possibilities we had, but we really didn't have any choice. I managed to ask if the baby was in any pain, and she couldn't answer that, but it most likely didn't have any pain … yet. She said that the baby wasn't “compatible with life” and that it would never survive outside of my stomach. If I continued the pregnancy, something which she did not advise, the head would grow so large that I couldn't give birth in the usual way. Kjersti, abortion, Hydrocephalus

A woman's own health may become an element in the situation. In Kjersti's case, this led to the doctor giving direct advice not to continue the pregnancy. An abortion can also prevent the foetus from suffering. The small word “yet” is important in the quote. The foetus will die anyway, and with an abortion a woman can take the initiative to solve a problem instead of delaying it. This indicates that a diagnosis of “incompatible with life” functions as a relief for women faced with the responsibility of making a decision. But does it always work like that, or does the diagnosis always lessen the dilemma that is represented by the pregnancy and abortion?

We decided very quickly that we would terminate the pregnancy. I felt we were strongly advised to do so. He said that it was incompatible with life. You can, of course, continue to term, but this child will surely die in your belly long before that. Plus, I had the beginnings of pre-eclampsia, which you often get with triploid babies (…) He said that it would be both a great risk for me … . *Did you consider the other possibility, then?* Yes, very much. I did. Since it sounded so brutal that I would get suppositories which would instigate labor. And he said that the child wouldn't survive the contractions because it was so little that it would die as a result of the contractions. And that I almost …, I didn't manage to think that thought. That I should take my child's life. Even though it would die later anyway. The thought that I would physically do something which stopped that heart which I could see was so strong, that was completely impossible for me to consider. (…) I felt that it was my choice, and that I wasn't forced, but that it was horribly painful to make it, and I didn't see any other solution. Couldn't see what the alternatives were. To go on until week 32, or 14 days, and go around the whole time thinking: is it alive or not? And how … Is this a painful life? I understood medically that it was the right thing to do. Also because I had begun to be sick. But it still seemed like an impossible thing to do. Sara, miscarriage, approved abortion, Triploid

Sara has problems with seeing how the medically correct can be possible, in spite of the potential “painful life” of the foetus in the womb. This is therefore a narrative about what the medical system *cannot* give Sara in her situation. Even though the doctor gives clear and justified advice, he cannot do anything about the fact that she must “take [her] child's life” and impose on the foetus a birth which will press the life out of it during contractions. As we have seen above, Sara experienced the relation to her baby as special and privileged, condensed in and symbolised by the strong heart, which she had to stop beating. Therefore the diagnosis offered no release, but rather an impossible situation in which there was no alternative to abortion, while the decision was nevertheless “horribly painful to make”. The duality is tangible. Also, in spite of the doctor's advice, there is no doubt about who has the authority to decide. Although the sovereignty is seen as a deep necessity, it is not at all experienced as a privilege, but on the contrary as a very heavy burden.

### Selective abortion: One's sacrifice for the child

I was fully ready when she said that she didn't know if it was in pain, I didn't want my child to suffer, especially not when it wasn't possible to save it. If there had been something we could have done to make it well, then maybe, but that was not the case. I have always been against abortion and never thought, I guess, that I would have to make an impossible choice like this. Kjersti, abortion, Trisomy 18

Because the condition is “incompatible with life”, the possibility of the child being in pain steers the decision to abort. Kjersti had “always been against abortion”, and therefore took responsibility for sparing the child by going against her own moral convictions, even though this made the choice “impossible”. This moral fracture amplifies the gravity of the situation. A picture is created of a mother who is willing to sacrifice her own norms and values in order for the foetus to escape suffering.

There was an immediately strong bond to the child. Which was threatened by something outside. Or inside. *Do you feel that, after you found out, that the bonds were stronger in those two days?* Yes, much stronger. Yes, very protectively compelled to be a mamma for the child, and that made it easier to manage a birth. Because I thought that it will be, in any case, the last thing I do for her. That she is born, that it doesn't happen any other way. That it is the last thing I can help her with, either here or there. Sara, miscarriage, approved abortion, Triploid

What we earlier saw Sara describe as “impossible”, a birth that will kill the foetus, is nuanced when it is understood as part of being a good mother. The special bond between the foetus and the pregnant woman, and in this case also between “mamma” and child, is further strengthened in the days before the abortion. This makes the abortion possible. It creates meaning, involving a foetus which is threatened and a mother who can protect and sacrifice. Through the sacrifice, the good mother gives something of importance to the foetus. In the narrative there is a coproduction of the ‘good mother' and ‘the good abortion'. We might even say that the meaning of the decision to have a selective abortion is displaced – from abortion to being a mother.

Those who have an abortion *for the child* protect it against pain, and thereby become good mothers. The sacrifice creates the picture of a woman who would do anything for her child. She becomes an “appropriate aborter” (Rapp, 1999, p. 237). But within this understanding there also lies the implicit picture of another kind of abortion, which is not as legitimate. The others are ‘bad aborters'. The position as a ‘good mother' is dependent on the notion that the foetus is being spared meaningless suffering.

In the case of Klinefelter syndrome it is different. Klinefelter is a ‘mild' chromosome defect. A child with the syndrome can grow up and become an adult who has an intelligence within the normal range. An adult with Down syndrome is visibly affected, but one has to be very familiar with Klinefelter syndrome in order to differentiate those affected from others – and in some cases it is not possible even with this knowledge. This makes *Vilde*'s appropriation of the “good mother” and “appropriate aborter” position problematic:

I will never, at any time, if I choose to terminate, manage to forgive myself, I will never be able to, in good conscience know and believe or speculate that it was done because it was the best for the child. Vilde, abortion, Klinefelter syndrome

Vilde is hard on herself. She both criticises and accuses her own motivations and reasons for aborting. We should not, however, misunderstand. It does not automatically imply that Vilde would accept the same kind of accusations from anybody else, and that they would be in any position to judge. It mainly concerns her relation to herself, and how she cannot “manage to forgive” herself. When women make use of culturally recognisable and dominant moral characters such as the “good mother” and the “self-sacrificing care-giver”, as Vilde, Kjersti and Sara more or less explicitly do, it may be interpreted as part of a conversation with themselves. Therefore we should not intervene, but listen. Nor do the uses of these moral characters automatically imply any judgements of other women who have made similar or different abortion decisions.^12^ However, it is possible that it is important for Vilde to present a picture of herself as not being superficial, the self-criticism showing that she fully grasps the serious dimensions of her decision.

The next case is about the limitations of the mother's sacrifice:

And I am a very compassionate person, who will manage everything and provide care and … So what mom and dad were afraid of was that I would ignore what the doctors indirectly said to me and say that I will manage this and if the young one here is going to live thirteen hours, then it will have thirteen good hours with me. They supported me 100%, but … you have to think of yourself and your husband now. And my husband said just about the same. I think my husband was relieved by me making the final decision I did. That this is too difficult to give birth to. Marit, abortion, Trisomy 13 or 18

In this case, the perspective on the child is turned around. The focus is not on the child's future suffering, but on the “thirteen good hours” it could possibly have with its mother. Birth is therefore the best alternative for the child, and it is not the abortion but the giving birth to the child that represents the sacrifice for Marit. The quotation concerns a compassion which would have gone much too far, and the woman, as “a compassionate person”, must be held in check by doctors, her mother, father and husband, who would have shared the burden. There is an immediate danger that Marit should overestimate her own capabilities. Although several other people amplify the necessity of having an abortion, it is Marit herself who has the authority to make the decision, and until it is finally made, the others are nervous about the outcome and what she might do to herself.

### Love

Gerd decided to give birth to a child shown to have Down syndrome:

Those we had already told it to when they visited, they have used phrases like “you are really so brave”, “you are so tough”, and “you must be so strong”. But we didn't want to hear these things. Brave, strong and tough didn't have anything to do with the choice we made. The ones who chose to terminate are braver, I think. To go through that process sounds completely terrible to me. Our decision didn't have anything to do with us wanting to be brave, tough or strong, it was just pure love … which made the decision. And I said to my husband after I last spoke with you: “how can I explain to her what it was that made us make the decision we made. How can one explain love?” Because it is really only love which does it … Love of the child that already grew inside of us. Gerd, birth, Down syndrome after amnio

There is an undertone to the comments from the people who “visited” that giving birth to a child with Down syndrome contradicts normal reason. We can suspect assumptions about a difficult future family life with a child with Down syndrome, who will demand endless amounts of time, self-sacrifice and work. There is no doubt that the rational thing to do would have been to terminate, as do almost all who carry a foetus shown to have Down syndrome. But love of the child cuts this argument short and establishes its own specific counter-logic. Although love is difficult to explain, as it concerns pure emotions that are placed in opposition to reason and rationality, “love of the child” is capable of producing a very powerful discourse. In some ways it is defensive, but the love discourse effectively externalises other considerations and decision criteria. Its real strength shows itself in the statement that termination of the pregnancy, or acting against the love, “sounds completely terrible to me”. Love makes it obsolete to be “brave, strong and tough”, even if the situation is obviously very difficult. There is also another remarkable aspect. Love has a de-individualising effect, establishing a small collectivity of Gerd and her husband in the situation (Luhmann, 1982). She not only talks about “the choice we made” and “our decision”, but even about “the child that grew inside of us”. This stands in rather stark contrast to the other cases.

## Authority and Vulnerability

Each moral, political and legal order is founded on an underlying state of normalcy (Schmitt, 1985 [1922]). Women tell of how the discovery of defects in the foetus casts them into a borderline state where it is difficult to distinguish the real from the unreal. The nightmare, the world of film and ‘aliens', becomes an intermediary of the meaning. The women find themselves on the verge of madness, and ‘impossible' appears to be the best description of the situation and the dilemmas. The happy pregnancy falls apart, being replaced by an impermeable pain. It is also clear that the basis for the general normative criteria has broken down. They have not disappeared, but lost their validity in a situation defined by the discovery of defects in the foetus. When the fundament for a general principle is gone, there is constituted a psychological, social and political state of emergency. ‘It is the Sovereign who rules over the exceptional'. This has a double meaning. First, the Sovereign has the authority to determine if the underlying state of normalcy, which is the fundament for the validity of the normative and political order, is in place or not. This means that it is the Sovereign who declares a state of emergency. In the narratives, it is only the women with defects detected in the foetus who have access to this unique insight as to how the state of normalcy is dissolved and replaced by the unreal, impossible borderline state. Second, the Sovereign has the authority to make decisions during the state of emergency. After the discovery of defects, the women have an exclusive understanding of the situation and of what the abortion dilemma involves. This constitutes their sovereignty over the decision.

There emerges an inapproachable exclusivity in this state of emergency. Only women who have themselves experienced the detection of defects in their foetus can begin to understand what is at stake in this situation. The narratives employ known characters such as the ‘good aborter' and ‘good mother'. They point to general and dominant moral criteria for human behaviour. But it is up to the pregnant woman herself as to whether or not these characters will be roused and introduced into the situation at all. No one else can do this. And no one can object if these characters do not appear in a narrative. It is not possible for those on the outside either to judge or to criticise these women's presentation of themselves as a “self-sacrificing care-giver” – or anything else. Also, ultimately it is not possible for society to offer any fundamental help if the women condemn themselves and their choices.

At the same time, language faces its limits. The narratives are filled with unfathomable and obscure metaphors, and the women themselves relate that they are not capable of communicating the nature of their situation. It is, after all, an attempt to do something impossible. There is an interesting link between this situation and some central aspects of the condition of disability in society. In her book *Staring: How We Look*, which discusses the social and psychological dimensions of disability, Garland-Thomson (2009) makes an interesting distinction between staring and the gaze. The gaze has both obvious and subtle normalising effects, categorising people and drawing the line between normality and deviance. The gaze constantly introduces social and cultural order into the world of experience, carrying the weight of history, both of disability and normality in general. Staring, however, does the very opposite. Unwillingly we are almost hypnotised by ‘the staree' (a linguistic invention by Garland-Thomson, designating the person being stared at), and we are lost in her, although it is considered impolite to stare. Because we are overwhelmed and magnetised by the pure presence of the staree, we are simply unable to label or categorise her. Therefore, staring dissolves order and represents the moment of transgression. This is very close to what happens when a deviance is discovered in the foetus. In a metaphorical sense the mother is staring at the imagined disability of the child within her body. The ordinary categories explode or run wild, which signals that they are of no use to her in such a situation. The mother has no choice other than to stare, being a mental prisoner of the almost unnamable features of her foetus, and the very experience is transgressing the ordering effects of the concepts of disability and normality. And it is exactly in this condition of staring, of losing herself in the extraordinary nature of the staree, that the mother has to make the decision of whether or not to abort.

The combination of exclusive insight and partial communicative breakdown constitutes an exclusive subject position for pregnant women in the question of selective abortion (Foucault, 1985). Partners, family, friends and medical personnel can influence, give indirect advice or inform as to what most other women choose to do. But they do not really grasp the important dimensions of the situation, and therefore cannot possess any decisive authority.^13^ Because the possibility of establishing a communicative community – not to mention rationality (Habermas, 1984) – around the abortion decision is blocked in important ways, there emerges a Sovereign in this state of emergency.^14^

There is an ambivalent relation between our perspective and the conceptualisations of the women as ‘moral pioneers' who are in different ways mapping new and unknown terrain. Just to state the trivial and obvious: the field of PND and selective abortion is not new – in spite of the fact that the range of techniques is constantly expanding – and is therefore hardly calling for any pioneers in the classical sense. This is also the case on the normative and moral level. The political debates have been tense and long-lasting. Our analysis, however, indicates that despite the entirety of the problematic around selective abortion being morally disputed, and the fact that many see worrying general developments in this area, a woman's decision is unencumbered and therefore in important respects is unavailable for external, normative evaluation. There begins to appear something like a ‘Decisionism', where a pure decision is made. The abortion decision establishes its own immanent criteria that cannot be traced back to general perspectives transcending the situation, but rather emerges as an integrated part of the decision itself.^15^ This is an important dimension to the position as Sovereign. Therefore, the terrain is always and repeatedly new and unknown. There are, however, no moral maps being produced that can be used by other women later, and half of the role of a pioneer vanishes, namely, that of facilitating the access and orientation of those who follow.

The women are not just Sovereigns. They must ‘live with the decision for the rest of their lives'. This creates an extreme vulnerability for the consequences of choosing whether or not to abort. The women's bodies and souls are subject to their own decisions (Foucault, 1985). Their fate, deeply affected by the choice, is neither regulated nor protected by generally applicable legal or moral principles, but rather is delivered into a pure decisionism, even though it is their own. The discovery of defects in the foetus therefore transforms these women into something Agamben (1998) has conceptualised as “bare life”.

The pregnant women are directly and totally at the mercy of their own sovereign decision. It will inscribe itself on body and soul for large parts of the rest of their lives. They ‘can't go the rest of [their lives] and see children with Down syndrome' if they have themselves chosen to abort; they cannot manage the thought of pressing the life out of a foetus through induced labour; they cannot envision stopping the foetus' strong heart beat; they are afraid of being overwhelmed as a care-giver if they do not abort and are frightened that a continued pregnancy will only provide great pain for the child. Fundamental pain will almost inevitably result from the decision. The only partial exception is the love discourse, which appears to establish a type of protection. Even in cases in which the foetus has no chance of survival, the abortion decision is a life and death decision which affects the woman's biological and existential condition. In this manner, the women's relations to themselves are constituted by a relation between the Sovereign and ‘Homo Sacer', simultaneously occupying both ends of the continuum of authority and sacrifice in the state of emergency. This is a dual position, being marked by both extreme authority and vulnerability.

## Relation to Oneself: The Sacrifice

The situation after the discovery of defects in the foetus does not just produce a dilemma but a compulsion. The women cannot avoid the question of whether or not to abort. But they need to give the decision a meaning, even if it means doing so uphill and against all odds. The situation presents itself as fundamentally chaotic and meaningless. Nonetheless, the women struggle to create order and meaning. They mobilise tropes of the good mother, women's responsibility, the good aborter, the pregnant woman who sacrifices herself for the child, the avoidance of suffering, ‘following your heart' and all-encompassing love.

The theme of sacrifice seems to be central. Although it comes in several different versions, it is the mother, and not the foetus or the child, who is going to be sacrificed. Therefore, it is fundamentally a self-sacrifice that the women describe. In some cases the self-sacrifice is really made, and in others it is not. The real and potential self-sacrifices concern different dimensions. The self-sacrifices that are really made are the pregnant woman's moral principles and her special relation to the child, while the self-sacrifices that are too big and heavy concern her own health and well-being. The latter remain potential, and for good reasons. The theme of self-sacrifice seems to be most dominant when the decision ends in abortion. Abortion is in no way an easy escape, and it is a recurrent theme that the abortion is made for and not against the child. Women are often prepared to set aside their own emotional needs and general moral convictions in order to prevent the foetus from suffering. The good aborting mother, the good aborter and the responsible woman are subject positions that are related to, and depend on, the self-sacrifice. Therefore it represents a privileged meaning dimension, and a core element in the narratives. Even when the self-sacrifice is not made, it is equally dominant in the discourse on the reasons for the abortion. When the self-sacrifice, in some cases, appears to be too great to guide the decision, it happens only after weighty and ‘impossible' deliberations. It then becomes the limit that is ultimately impossible to transgress, although the woman may be very tempted to do so. In such cases the pregnancy may turn into a risk, where the threat is coming from the woman as sovereign decision maker. In *Vilde*'s case, the very lack of self-sacrifice is turned into self-accusations, self-inflicted suffering and pain of not accepting her justifications for the decision as being good enough – as if it is taking a moral revenge on her.

At the same time, there is a related message on another level. Fighting a battle involving all of one's emotions becomes an unavoidable part of the process. Nothing can be concealed. The most brutal words must be used. By asking questions concerning one's own sanity and the humanity of the foetus, it is revealed that the women's relation to themselves finds itself on the ‘borderline'. It is almost too much, and we get the impression that they are not far from a nervous breakdown. The emotional involvement becomes so powerful that it can border on madness. In a way it is communicated that the women have already been sacrificed by the very situation into which they have been brought by the pregnancy, the detection of serious defects in the foetus and the compulsion to make a fast, and in the end lonely, decision on whether or not to abort. This is different from the self-sacrifice. It is not deliberate or chosen, but unavoidable and a fundamental dimension of their existential condition. The consequence on the normative level, however, is that they become immune against any kind of critique from other people or institutions. It is not possible to pass any moral judgements on a person who already has been sacrificed. With the exception of *Vilde*, it also protects against self-criticism. In some ways, the women are outside the realms and space of ordinary, normative order – not only as sovereigns, but also as sacrificed.

The impacts of the sacrifices are especially felt by the women who decide to abort. It is not limited to them, but it seems as if the women who do not abort are somehow protected or shielded from the effects. This is partly related to their reasons, or more precisely their experiences of the forces present in the situation. For *Gerd*, there was – on a certain level – no decision made about abortion. The understanding of love lends itself to a belief in destiny. It is not chosen, but rather affects people. Love chooses people, not the other way round, and thus creates a fundamental necessity. It was love of the child that “made that decision”, and the necessary strength lay neither with the woman nor with her husband, but with love. In this way, the decision was lifted from the shoulders of the woman and her husband, and given to the power that possesses them.

*Gerd* has a strong orientation towards the difficulties and challenges of the life together with the child with Down syndrome after birth: “Yes, and so we are not afraid of the challenges any of us, and both share the belief that we do not receive more than we can endure … ”. But even this is framed by a belief in fate. Existence gives them only that which they can manage. Gerd thus communicates that there lies a greater reason behind these events, which is inscrutable.

There are hardly any explicit Christian or religious motifs and arguments present in our interviews, unless as negations such as “I am not a religious person”. We must, however, be aware of the different levels of the discourses. Behind the secular veil there are some Christian figures that are not easily overseen.^16^ The sacrifices in different versions are the most obvious.^17^ It is also possible to make arguments in favour of the love discourse having, at least partly, religious origins. In this context it is also tempting to recall Schmitt's argument that most of the important political concepts are secularised religious concepts, including that of the Sovereign. Nevertheless, the severity and harsh reality of the field of selective abortion does not lend itself easily to any kind of superficial everyday rationality. Other dimensions come inadvertently to the foreground, and we tap into some deeper levels of the cultural discourses.^18^ However, in this context we can only make some very short and preliminary indications of these.

## A Final Remark

Our analysis shows a central paradox. At the core of the social field of selective abortion lies a series of states of emergency. These arise continuously, thereby producing a stable situation. The states of emergency define society's normal relation to the decisions of selective abortion, and constitute the most fundamental aspects of regulation in this area. The state of emergency and the state of normalcy therefore blend together. The distinction itself is undermined, and its own ‘zone of indistinction' appears around the abortion decision. This characterises the legal, political and normative regimes in the field.

## Footnotes

1 There is a very heated ethical debate regarding the nature of this link. Shakespeare (2006) discusses the so-called “expressivist objection” to PND and concludes: “Nor should we interpret a decision to have a test or a termination as expressing disrespect or discrimination towards disabled people” (p. 102). For a critique of Shakespeare's position, see Holm (2008).

2 The study was done with financial support from the Norwegian Research Council.

3 This is partly because genetic counselling is meant to be normatively neutral. Medicine's conception of ‘the patient's best interests' is replaced with information on the range of possible choices. Markens (2013) has interviewed genetic counsellors in the United States about their views on genetic testing, with a particular emphasis on prenatal testing. They generally have positive attitudes, underlining the ‘empowerment' of women, widening the range of choices and supporting ‘health management'. Although there might be some cultural differences between Norway and the United States, our short analysis indicates that there are some other important dimensions present.

4 Gupta interviewed the women and their husbands after they had received councelling on the basis of risk profiles, but before they had amniocentesis. Although there were good practical reasons for this choice, there are some methodological fallacies associated with it.

5 The tests are biochemical analyses of maternal blood, and give an individual risk for Trisomy 13, 18 and 21 and ‘open defects' (triple test). The risk assessment is calculated on the basis of these tests, ultrasound results and the mother's age.

6 In our material we do not find any traces of the aggressive discourse of hope, which is prevalent among many patient groups, as well as on the political level, in relation to the new genetics and biomedicine (Brekke and Sirnes, 2011). There is, however, an interesting parallel concerning the sovereignty of suffering, which has important implications for the normative regime.

7 We are referring to where there is social disagreement over whether a certain diagnosis will be the cause of great pain and suffering for the future child or family. Klinefelter syndrome can be an example.

8 “The ‘it' represented by a positive outcome from the genetic testing for disability becomes a signification of what can go ‘wrong' with a fragile humanity. Thus, the ‘girling' of the ‘girl' (or the ‘boying' of the ‘boy') is overshadowed by the ‘marker' disability and its present and future implications” (Overboe 2007).

9 Women experience a new recognition on two levels during ultrasound examinations. They first experience the realisation that the pregnancy is true and real: “The other is that this fetus is a human, another human, that she has a body that is not only her own” (Ravn, 2004, p. 264).

10 The films thematise reproduction, birth and the relationship between the human and the alien, or animal (Constable, 1999).

11 Temporality is a complex issue. It is present in our cases in different ways, and there are several paradoxes. Although there is an extraordinary legal regime of selective abortions, the medical system to some degree signals that time is more pressing than it actually is, and this affects the women's experiences of the situation. Also, the pace of the process is partly governed by the perceived needs of the women, for instance whether they want to terminate as soon as possible or whether they need some extra time to come to terms with their relation with the affected foetus. Generally speaking, there is a socio-psychological paradox present. Although the women may feel compelled to make the abortion decision within a couple of weeks, several of them seem to enter a situation of pure presence, or an extraordinary condition where the time dimension as such is dissolved.

12 Rapp (1999, p. 237) touches upon the same problematic when she describes how women who have had a selective abortion see and evaluate the “blind” abortions: “Yet I was forced to recognise the *cultural* judgment that many women with positive diagnoses make about their own circumstances. They considered themselves to be appropriate mothers, and therefore, tragically, appropriate aborters. While they often expressed compassion for teenage girls “in trouble”, they did not want to be identified with them in any social sense”. On the basis of our material, we have a somewhat different interpretation.

13 Schwennesen and Koch (2012) present the socio-ethical argument that as all prenatal knowledge is socially constructed, the health personnel should both admit and embrace their influence by offering some advice to the pregnant women. Then they would provide ‘good' care, built on a realistic understanding of their unavoidable power. Schwennesen and Koch concentrate on the question of further testing after the presentation of individual risks. In the situation of abortion decision, however, the socially constructed categories tend to dissolve, and thereby also the above potential for good care. Also, Schwennesen and Koch seem to invite health personnel to a strategic communication about the foetus, which in our view would be inadvertently revealed in the situation. If a woman realises that a main intention of the communication is to comfort her, the effect might be the very opposite.

14 This is also clear from a legal perspective. Even though selective abortion is a free choice only up to the 12th week of pregnancy, and the mild regime which emphasises women's subjective evaluations and wishes is replaced by a much stricter set of laws after week 18, it is clear that the normal structure of law does not hold for selective abortion. Jurisprudence abdicates in favour of a flexible ‘real-legality', or rather, exceptional regulation which respects women's position as Sovereign. Selective abortion takes place flexibly long after the 20th week, and meetings with the abortion committees are not marked by reassessments.

15 This marks the limits of the pregnant woman as an individual manager being caught by the mental world of probabilities and abstract, statistical risk categories (Samerski, 2009). The decision criteria of this kind of rationality dissolves in the microstate of emergency. They might exercise a considerable socio-psychological power during the phases of prenatal testing, but not in the decision situation, after something serious is detected.

16 In her article about selective abortion in Japan, Kato (2009) has some interesting examples of the influence of Buddhist dimensions.

17 The logic of sacrifice is far removed from the responsible management of health and life based on expert advice, which according to Samerski (2009) dominates the reasoning of women in the field of selective abortion. The sacrifice may potentially be very self-destructive and irresponsible, although its limitations may of course depend on a logic of responsibility. The most important factor, however, is that the sacrifice belongs to a very different dimension of experiencing and thinking. Yet there are some subtle elements in Samerski's perspective that point in this direction, especially when she discusses the meaningless and impossible choices imposed on the pregnant women, dissolving the very possibility of acting in a managerial way. See also Rose (1999).

18 There is potentially an uneasy relation between our perspective and some feminist traditions. The role of feminism within bioethics is a vast question, but we will just indicate that there might be some affinities between the logic of sacrifice and the so-called third-wave feminism (Snyder, 2008). Although they concentrate on sexuality (which is not far from the questions of reproduction and abortion), the third-wave feminists embrace diversity, paradoxes and cultural figures which seemed to be oppressive from the second-wave perspective. Although they lack comprehensive power analyses, they realise that these should not be done prematurely and superficially.
